# Clinical and prognostic differences in oropharyngeal squamous cell carcinoma in USA and Denmark, two HPV high-prevalence areas

**DOI:** 10.1016/j.ejca.2024.113983

**Published:** 2024-03-02

**Authors:** Amanda-Louise Fenger Carlander, Simone Kloch Bendtsen, Jacob H. Rasmussen, Kathrine Kronberg Jakobsen, Martin Garset-Zamani, Christian Grønhøj, Jeppe Friborg, Katherine Hutcheson, Faye M. Johnson, Clifton D. Fuller, Amy C. Moreno, Toyin Babarinde, Neil D. Gross, Jeffrey N. Myers, Christian von Buchwald

**Affiliations:** a Department of Otolaryngology, Head and Neck Surgery & Audiology, Copenhagen University Hospital - Rigshospitalet, Copenhagen, Denmark; b Department of Oncology, Copenhagen University Hospital - Rigshospitalet, Copenhagen, Denmark; c Department of Head and Neck Surgery, Division of Surgery, The University of Texas M.D. Anderson Cancer Center, UTMDACC, TX, USA; d Department of Thoracic Head and Neck Medical Oncology, The University of Texas M.D. Anderson Cancer Center, UTMDACC, TX, USA; e Department of Radiation Oncology, The University of Texas M.D. Anderson Cancer Center, UTMDACC, TX, USA; f Department of Head and Neck Surgery, The University of Texas M.D. Anderson Cancer Center, UTMDACC, TX, USA; g The University of Texas Graduate School of Biomedical Sciences; UTMDACC, TX, USA

**Keywords:** Human papillomavirus, Oropharyngeal cancer, Squamous cell carcinoma, Survival, Recurrence, Prognosis, Prevalence, Demographics

## Abstract

**Background:**

Uncertainty persists regarding clinical and treatment variations crucial to consider when comparing high human papillomavirus (HPV)-prevalence oropharyngeal squamous cell carcinoma (OPSCC) cohorts for accurate patient stratification and replicability of clinical trials across different geographical areas.

**Methods:**

OPSCC patients were included from The University of Texas MD Anderson Cancer Center (UTMDACC), USA and from The University Hospital of Copenhagen, Denmark from 2015–2020, (n = 2484). Outcomes were 3-year overall survival (OS) and recurrence-free interval (RFI). Subgroup analyses were made for low-risk OPSCC patients (T1–2N0M0) and high-risk patients (UICC8 III-IV).

**Results:**

There were significantly more HPV-positive (88.2 % vs. 63.1 %), males (89.4 % vs. 74.1 %), never-smokers (52.1 % vs. 23.7 %), lower UICC8-stage (I/II: 79.3 % vs. 68 %), and fewer patients treated with radiotherapy (RT) alone (14.8 % vs. 30.3 %) in the UTMDACC cohort. No difference in the adjusted OS was observed (hazard ratio [HR] 1.21, p = 0.23), but a significantly increased RFI HR was observed for the Copenhagen cohort (HR: 1.74, p = 0.003). Subgroup analyses of low- and high-risk patients revealed significant clinical and treatment differences. No difference in prognosis was observed for low-risk patients, but the prognosis for high-risk patients in the Copenhagen cohort was worse (OS HR 2.20, p = 0.004, RFI HR 2.80, p = 0.002).

**Conclusions:**

We identified significant differences in clinical characteristics, treatment modalities, and prognosis between a Northern European and Northern American OPSCC population. These differences are important to consider when comparing outcomes and for patient stratification in clinical trials, as reproducibility might be challenging.

## 1. Introduction

Human papillomavirus (HPV) is a key factor in the rising incidence of oropharyngeal squamous cell carcinoma (OPSCC), but with geographical variation [1–4]. How HPV-positivity is defined varies, but the expression of p16 is widely accepted as a surrogate marker for HPV-positivity. However, double p16/HPV-positivity more accurately classify biologically active HPV infection, better predicts patient outcomes, and standardized HPV testing has been recommended [4,5].

Given the remarkable treatment response [6] and the toxicities associated with treatment [7], attempts have been made to identify low-risk HPV-positive (HPV+) patients suitable for treatment de-intensification [8,9]. Some well described factors associated with low-risk OPSCC include limited smoking history, p16 + status, and low tumor burden [6].

Both the U.S. and Denmark are HPV high-prevalence areas [10,11]. However, the healthcare systems in Denmark and the U.S. exhibit significant differences. Denmark operates under a public, universal healthcare system and all cancer diagnoses, treatments, and follow-ups are conducted with the standardized cancer care packages available within the public health care system. The healthcare system in the U.S. is larger and more complex, with patients being treated at private or public care centers based on a variety of factors, including access to health insurance.

The consideration of potential clinical and demographic variations is crucial when interpreting the prognosis across different patient populations, for accurate patient stratification and management and to ensure reproducibility and replicability of clinical trials in diverse geographical areas.

The aim of this study was to identify potential differences in clinical characteristics, treatment modalities given, and prognosis among OPSCC patients in two HPV high-prevalence areas with distinct populations and health care systems: The Copenhagen Oropharyngeal Cancer Database (COHOC) at the University Hospital of Copenhagen, Denmark and the Stiefel OPSCC Database at The University of Texas MD Anderson Cancer Center, USA (UTMDACC). Second, we wished to identify if such differences impact the prognosis for a subgroup of low-risk (T1T2N0M0) and high-risk (III-IV) patients potentially eligible for de-escalated or intensified therapy, respectively.

## 2. Materials and methods

### 2.1. Study design, setting and population

This retrospective cohort study was approved by The Regional Scientific Ethical Committee (H-20072877) and the Danish Data Protection Agency and followed the Strengthening the Reporting of Observational Studies in Epidemiology (STROBE) reporting guideline [12]. The Stiefel OPSCC Database is approved by The Institutional Review Board of the University of Texas MD Anderson Cancer Center (UTMDACC) (protocol PA 14–0947).

The study cohort consisted of all patients with a new OPSCC diagnosed from 2015–2020 treated with curative intent from two independent OPSCC cohorts: *The COHOC Database* is a population-based, retrospective cohort, comprising patients diagnosed with OPSCC from 2000–2020 in Eastern Denmark and was previously well described [13–15]. *The Stiefel OPSCC Database* is a prospective, longitudinal cohort initiated at UTMDACC. Since 2015, 1671 OPSCC patients have been enrolled with detailed characterization of exposures, HPV-status, diagnostic/staging, treatment, disease control, and longitudinal collection of validated clinician- and patient-reported survivorship outcomes from diagnosis through 5-years of follow-up.

### 2.2. Variables

The primary outcomes were 3-year overall survival (OS) and 3-year recurrence-free interval (RFI). OS was defined as time from diagnosis to death of any cause and patients alive were censored at the last date of follow-up or 3 years after diagnosis. RFI was defined as the time from primary diagnosis to the diagnosis of recurrence (locoregional and/or distant) and patients without recurrence were censored at last day of follow-up, date of death or 3-years after diagnosis. HPV-positivity was defined as being positive for both HPV DNA and p16 in the Copenhagen cohort, while HPV-positivity was defined as being positive to HPV or p16 in the UTMDACC cohort. Details on HPV detection, p16 immunohistochemistry, and measurements are provided in Supplement 1.

### 2.3. Treatment

Therapeutic decisions were made during a multidisciplinary treatment planning conference based on factors such as disease stage, anatomical subsite, comorbidity, functional considerations, and the preferences of both patients and clinicians. In Denmark, treatment decisions are in accordance with the Danish Head and Neck Cancer Group (DAHANCA) national guidelines [16,17], while treatment decisions generally follow the National Comprehensive Cancer Network (NCCN) guidelines at UTMDACC [18]. Details on treatment guidelines are provided in Supplement 1.

### 2.4. Statistics

Statistical analysis was performed in R statistics version 4.1.3. Continuous variables were reported as median values with an interquartile range (IQR) and categorical variables as frequencies. To test for significance, Pearson’s chi-square test was used for the binomial categorical covariables, Fisher’s exact test was used for small sample sizes, and a *t*-test was used for the quantitative covariables distributed in two groups. We considered a *p*-value < 0.05 to be statistically significant. Age and follow-up time were included as continuous variables and the other variables were considered categorical variables.

OS and RFI were evaluated by Kaplan–Meier curves and with the log-rank method and by uni- and multivariable Cox regression analyses (packages Survminer and Survival). For the total OPSCC cohort, the OS (372 events) and RFI (267 events) multivariable analyses were adjusted for treatment center, age (cut-off >60 years), gender, HPV-status, smoking status, tumor location, T-, N- and M-class and treatment modality. For model control, proportionality was tested with Score residuals. Details on subgroup analyses are provided in Supplement 1.

## 3. Results

### 3.1. Patient characteristics

A total of 2484 patients were included, 1216 patients from Eastern Denmark and 1268 from UTMDACC. The median follow-up time was 2.1 years (IQR:1.0–3.6) in the UTMDACC cohort and 2.3 years (IQR: 1.7–3.3) in the Copenhagen cohort, p < 0.001. The UTMDACC cohort included significantly more men (89.4 % vs. 74.1 %), younger age (median age 60 vs. 62 years), and never smokers (52.1 % vs. 23.7 %). See Table 1.

### 3.2. Clinical tumor characteristics

In the UTMDACC cohort, the predominant tumor site was the base of tongue (BOT) (48.6 %) followed by the palatine tonsils (47.2 %) while the palatine tonsils were the predominant tumor site in the Copenhagen cohort (51.6 %). OPSCC patients presented at the UTMDACC at significantly lower T-stage (T1-T2: 72.9 % vs. 64.9 %), lower N-stage (N0-N1: 76.8 % vs. 73.3 %) and lower UICC stage (I-II: 78.4 % vs. 69.2 %) than in Copenhagen. See Table 1.

### 3.3. HPV status

Overall, 63.1 % of the OPSCC patients were HPV+ in the Copenhagen cohort, while 88.2 % were HPV+ in the UTMDACC cohort. In the UTMDACC cohort, 68.9 % did not have both HPV and p16 status available, while this was the case in 3.1 % of the patients in the Copenhagen cohort. In the Copenhagen cohort, 0.4 % and 10.8 % in the UTMDACC were tested for HPV DNA only, while 1.9 % in the Copenhagen cohort and 46.7 % in the UTMDACC cohort were tested for p16 only. See Table 1.

### 3.4. Treatment modalities

Radiation-based treatment was predominant in both cohorts, but significantly more patients received radiotherapy (RT) alone in the Copenhagen cohort (30.3 % vs. 14.8 %). In the Copenhagen cohort, 51.0 % received concurrent chemotherapy (CRT) vs. 49.8 % in the UTMDACC cohort. Neoadjuvant chemotherapy + RT/CRT was given to 16.7 % of patients in the UTMDACC cohort. Significantly more patients received surgery alone in the Copenhagen cohort (16.0 % vs. 8.7 %), while significantly more received surgery and post-operative adjuvant therapy in the UTMDACC cohort (9.8 % vs. 2.7 %). See Table 1.

### 3.5. Overall survival

**Fig. 1.**
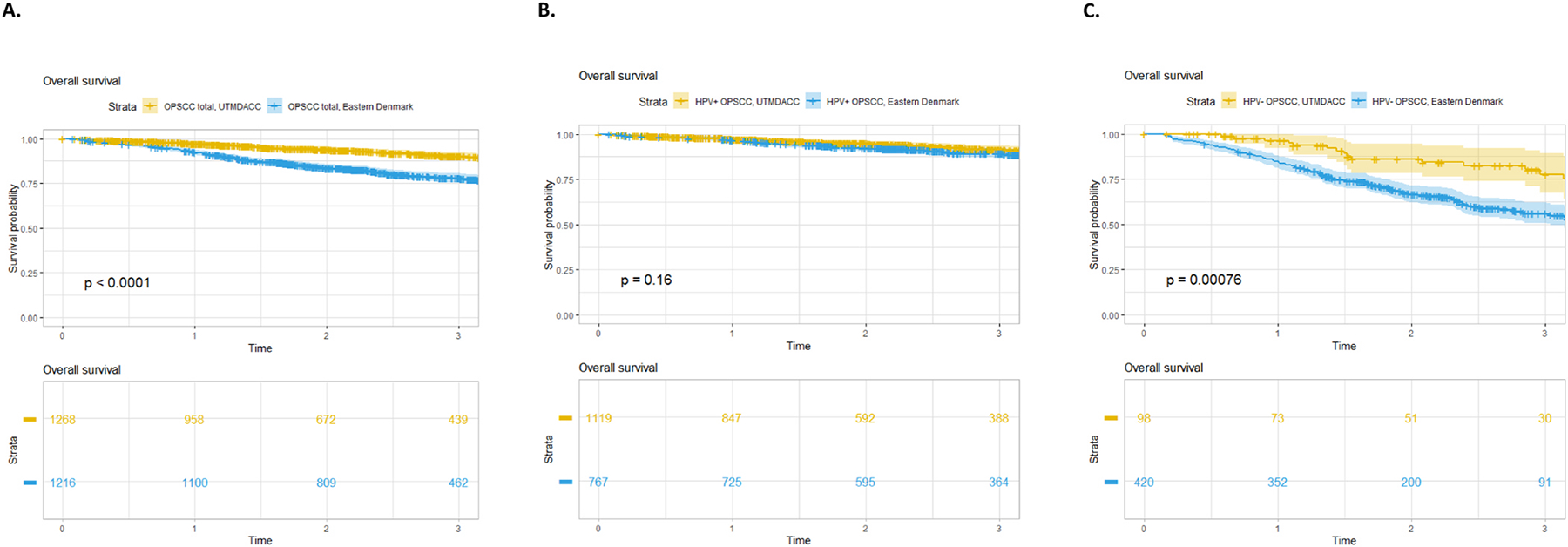
Kaplan-Meier curves depicting the overall survival probability stratified by center and HPV status from 2015–2020. A. All OPSCC 2015–2020 stratified by center. B. HPV+ OPSCC stratified by center. C. HPV− OPSCC stratified by center. *Abbreviations:* UTMDACC, The University of Texas MD Anderson Cancer Center, USA, HPV, human papillomavirus; OPSCC, oropharyngeal squamous cell carcinoma.

Kaplan-Meier OS models showed significant differences in the 3-year OS for the UTMDACC cohort (91 % [95 % CI: 89–93 %]) compared to the Copenhagen cohort (83 %, [95 % CI: 80–85 %], log-rank p < 0.001) which was also noted for HPV-negative (HPV−) patients. See Fig. 1 and supplementary Table 2 (Supplement 1).

The multivariable OS analysis revealed no significant difference between the two cohorts (hazard ratio [HR] 1.21, p = 0.23). A significantly increased HR was observed for HPV−, current smoking, T3- and T4-class, N2 and N3-class, M1-class, and RT. See Table 2.

### 3.6. Recurrence-free interval

**Fig. 2.**
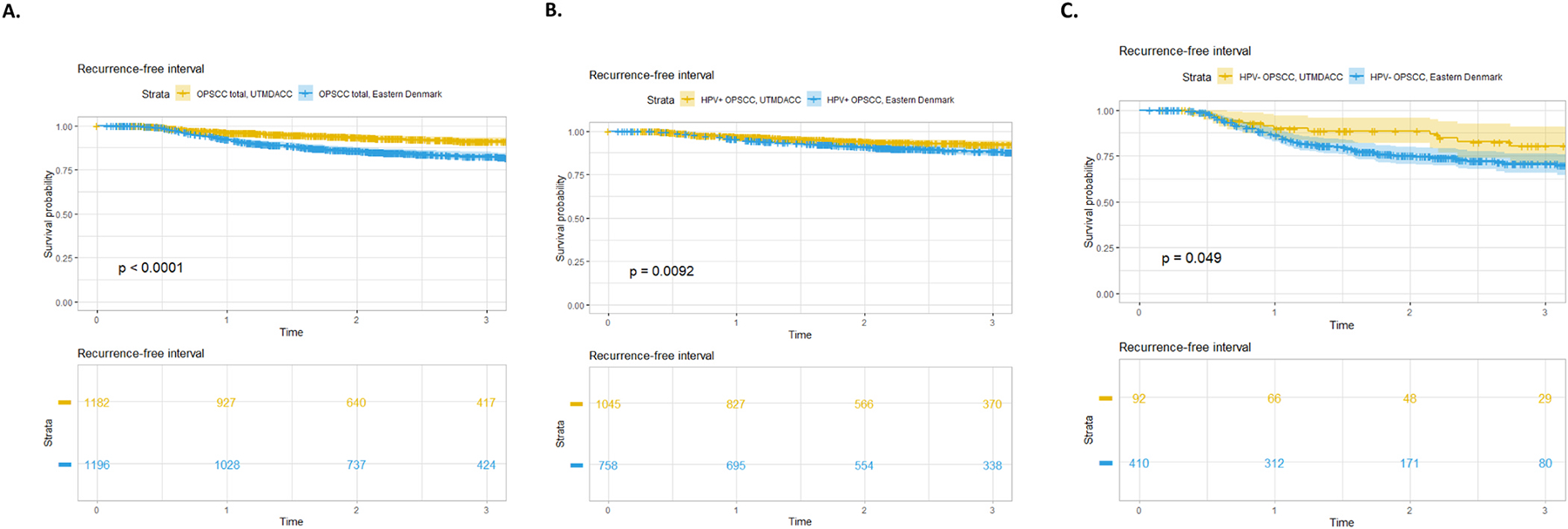
Kaplan-Meier curves depicting the recurrence-free interval stratified by center and HPV status from 2015–2020. A. All OPSCC 2015–2020 stratified by center. B. HPV+ OPSCC stratified by center. C. HPV− OPSCC stratified by center. *Abbreviations:* UTMDACC, The University of Texas MD Anderson Cancer Center, USA, HPV, human papillomavirus; OPSCC, oropharyngeal squamous cell carcinoma.

Overall, 83 OPSCC patients had a recurrence in the UTMDACC cohort, with a median time to recurrence of 1.00 years (IQR: 0.63–1.91 years). In the Copenhagen cohort, 184 had a recurrence with a median time to recurrence of 1.04 years (IQR: 0.69–1.61). The 3-year RFI was significantly better in the UTMDACC cohort (91.3 % [95 % CI 89.3–93.3 %]) compared to the Copenhagen cohort (82.7 % [95 % CI 80.3–85.1 %], log-rank p < 0.001), which was also found when stratifying for HPV-status. See Fig. 2.

The multivariable recurrence analysis showed a significantly increased HR in the Danish cohort (HR: 1.74, p = 0.003). Also, HPV−, T2-T4-class, N1-N3-class, M1-class, RT and neoadjuvant chemotherapy + RT/CRT had a significantly increased HR. See Table 3. The analysis was also performed for patients with either progression or second primary tumors, which did not significantly impact the conclusions; data not shown.

### 3.7. Subgroup analysis of low-risk patients with T1/T2N0M0 OPSCC

The UTMDACC T1T2N0M0 cohort exhibited significantly more men, younger ages, more never smokers, more HPV+, fewer OPSCC from other locations, and more received CRT than the Danish T1T2N0M0 cohort. See Table 3 (Supplement 1). In total, 26 had a recurrence with a median time to recurrence of 1.07 years (IQR 0.82–1.47) and 0.76 years (IQR 0.63–1.75) in the Copenhagen and the UTMDACC T1T2N0M0 cohorts, respectively.

Kaplan-Meier models showed a significantly higher 3-year OS in the UTMDACC T1T2N0M0 cohort (89.9 % vs. 79.3 %, log-rank p = 0.01) and 3-year RFI (96.2 % vs. 86.5 %, log-rank p = 0.011). See Fig. 1 (Supplement 1). These differences were not reproduced in the multivariable analysis for both OS and RFI. See Table 4 and 5 (Supplement 1).

### 3.8. Subgroup analysis of high-risk patients with stage III-IV OPSCC

The UTMDACC III-IV cohort exhibited significantly more men, younger age, more never smokers, more HPV+, fewer other OPSCC locations, higher UICC-stage for HPV+ and fewer received RT. See Table 6 (Supplement 1). In total, 126 had a recurrence with a median time to recurrence of 0.88 years (IQR 0.59–1.32) and 1.19 (IQR: 0.54–1.71) in the Copenhagen and the UTMDACC III-IV cohorts, respectively.

Kaplan-Meier models showed a significantly higher 3-year OS in the UTMDACC III-IV cohort (77.1 % vs. 52.4 %, log-rank p < 0.001) and 3-year RFI (78.6 % vs. 65 %, log-rank p = 0.001). See Fig. 2 (Supplement 1). These differences were reproduced in the multivariable analysis for both OS (HR 2.20, p = 0.004) and RFI (HR 2.80, p = 0.002). See Table 7 and 8 (Supplement 1).

### 3.9. Subgroup analysis of RT single modality patients

See Results (Supplement 1).

## 4. Discussion

This study, which included 2484 patients with OPSCC from Eastern Denmark and UTMDACC treated between 2015–2020, revealed significant differences in OS and RFI driven by clinical and treatment differences.

Although RT was predominant in both cohorts, significantly more patients received RT alone in the Copenhagen cohort, which is noteworthy, since the Copenhagen cohort presented at higher UICC-stage, T- and N-class. In line with this, RT remains the predominant treatment modality in most European centers [19].

The UTMDACC cohort included more clinical characteristics associated with a better prognosis: more HPV+ OPSCC patients, lower T- and N-burden and less smoking [20] but also more men which are associated with a worse prognosis [21]. When adjusting for these differences, an independent increased OS HR was not observed, but the risk of recurrence was significantly higher in the Copenhagen cohort (RFI HR 1.74, p = 0.003). Discrepancies in follow-up regimens and divergent definitions of recurrence may also attribute to the observed disparities. Although speculative, it could also indicate a different biology of HPV+ OPSCCs. In line with our findings, a recent study including a Danish and a Toronto cohort observed significant clinical and treatment differences, which were reflected in differences in locoregional failure and OS [22].

In low-risk patients, significant clinical and treatment differences were observed with 41.7 % in the Copenhagen cohort receiving RT alone versus 19.6 % in the UTMDACC cohort, yet the prognosis remained equally good. Several studies have investigated de-escalated treatment to minimize treatment-related toxicities for a subgroup of patients with HPV+ OPSCC [8,9,23–25]. It is important to keep in mind that substantial clinical differences exist when selecting patients and validating de-escalating trials in various geographical areas. This study underlines the importance of including multiple factors like TNM-classification, HPV− and smoking status, but also indicates that factors like age and tumor location could be considered for stratification.

Conversely, a sub-analysis of high-risk patients revealed significant clinical and treatment differences influencing the prognosis. The UTMDACC cohort had significantly better OS and RFI. OS was also significantly associated with current smoking, stage, and treatment modality given. RT alone was given to a substantial part of the Copenhagen patients (37.1 % vs 7.2 %), despite that CRT is recommended for this group [16,17]. The difference in use of concurrent chemotherapy is likely a result of factors impacting treatment selection not included in this study, e.g., comorbidities, as the Copenhagen cohort is older and include more smokers. But a difference in practice patterns cannot be excluded. Neoadjuvant chemotherapy is not used in Denmark but was given to 16.7 % of the patients in the UTMDACC cohort (corresponding to 39.1 % of UTMDACC high-risk patients), which is in line with data from The National Cancer Database [26]. The role of neoadjuvant therapy in locally advanced OPSCC cancer remains controversial [27], however, it may be associated with a decreased risk of distant metastasis [28]. A recent study suggests that neoadjuvant therapy is associated with an improved OS and lower risk of distant metastasis in patients with OPSCC [29].

A notable difference in treatment between MDA and Denmark is the use of hypoxic modification with nimorazole. The DAHANCA 5 randomized trial, investigated the effect of nimorazole, showed a significantly better loco-regional control rate and lower cancer-related death in patients receiving nimorazole compared to placebo [30]. Nimorazole has since been standard of care for all Danish OPSCC patients. The benefit of nimorazole may primarily be present in HPV− OPSCC, as reanalysis showed no significant benefit in HPV/p16-positive tumors [31].

Disparities in the healthcare system might introduce a selection bias contributing to the differences observed in this study, with more HPV-OPSCC patients and more high-risk patients in the Copenhagen cohort. Studies have shown that insurance status is predictive of clinical characteristics such as tumor stage and comorbidities at diagnosis as well as oncological outcome [32–34]. A recent Copenhagen study comprising a part of the Copenhagen cohort suggests that low socioeconomic status negatively impact the OS, largely due to differences in clinical characteristics at diagnosis, including smoking status, comorbidities, and clinical stage [35]. Future studies including socioeconomic status, comorbidities, and performance status would provide further insight into this matter. Disparities in the healthcare system might also contribute to the differences observed in treatment modalities, availability, and regimen of follow-up care.

### 4.1. Limitations

A recent study, where the Copenhagen cohort comprised the majority of the study cohort, has proven double HPV/p16-positivity to be superior in prognostication and has led to the recommendation of including both p16 and HPV-status in areas with high discordance, which is also supported by the ESMO guidelines [4,5]. In this study, HPV-positivity was defined in the UTMDACC as p16 + or HPV+, which might overestimate the HPV-positive prevalence, although only 1.9 % had discordant p16/HPV status [4]. The included cohorts might not be representative of the Northern American and European cohorts overall, and the results may not apply to other regions. Socioeconomic status, performance score, and comorbidities were not included which potentially impacts treatment decisions and have shown to influence both OS and RFI and which we hypothesize differs greatly in the two cohorts.

## 5. Conclusion

Significant demographic, clinical, and treatment differences influencing the prognosis were identified between two high HPV-prevalence OPSCC cohorts representing two different health care systems: a Copenhagen cohort from a public, universal health care system and a UTMDACC cohort from an insurance-based health care system. Despite significant clinical and treatment differences, the prognosis for low-risk OPSCC patients was equally good, while the prognosis was significantly better for patients with high-risk OPSCC in the UTMDACC cohort.

Our study suggests that significant clinical differences exist between a Northern European and Northern American OPSCC population, which is important to consider when comparing outcomes and for patient stratification in clinical trials, as reproducibility might be challenging. However, to fully understand possible intercontinental variations, more data are needed.

## Supplementary Material

Appendix A: Supplementary material

## Figures and Tables

**Table 1:** Characteristics of 2484 patients with OPSCC in Eastern Denmark and UTM-DACC, USA from 2015–2020.

Variable	Eastern Denmark. n = 1216		UTMDACC, USA. n = 1268		
			
	no.	%	no.	%	*P-value* ^a^

**Gender**					< 0.001^a^
Male	901	74.1	1133	89.4	
Female	315	25.9	135	10.6	
**Median age (IQR)**	62 (56–70)		60 (54–67)		*< 0.001* ^a^
no.	1216		1268		
**Median follow-up. years (IQR)**	2.3 (1.7–3.3)		2.1 (1–3.6)		*< 0.001* ^a^
no.	1216		1210		
**Smoking**					*< 0.001* ^a^
Current	464	38.2	66	5.2	
Former	458	37.7	535	42.2	
Never	288	23.7	661	52.1	
Unknown	6	0.5	6	0.5	
**HPV-status**					*< 0.001* ^a^
HPV+	767	63.1	1119	88.2	
HPV−	420	34.5	98	7.7	
Unknown	29	2.4	51	4	
**HPV/p16-status**					*< 0.001* ^a^
HPV+/p16+	767	63.1	345	27.2	
HPV+/p16−	49	4.0	8	0.6	
HPV−/p16+	35	2.9	18	1.4	
HPV+/p16−	49	4.0	8	0.6	
HPV−/p16−	327	26.9	23	1.8	
Unknown	38	3.1	874	68.9	
**HPV genotype**					*< 0.001* ^a^
HPV16	662	87.8	109	87.9	
HPV33	59	7.8	1	0.87	
Other genotypes	33	4.4	14	11.3	
**Tumor location**					*< 0.001* ^a^
BOT	374	30.8	617	48.7	
Tonsil	628	51.6	599	47.2	
Other	214	17.6	52	4.1	
**TNM Stage (UICC/AJCC 8), HPV+**					
**T-class**					*0.005* ^a^
T1	241	31.4	400	35.7	
T2	289	37.7	433	38.7	
T3	116	15.1	168	15.0	
T4	119	15.5	118	10.5	
Unkown	2	0.3	0		
**N-class**					*< 0.001* ^a^
N0	121	15.8	125	11.2	
N1	514	67.0	768	68.6	
N2	103	13.4	203	18.1	
N3	29	3.8	23	2.1	
Unknown	0		0		
**M-class**					*0.01* ^a^
M0	762	99.3	1093	97.7	
M1	2	0.3	14	1.8	
Unknown	3	0.4	12	1.1	
**Overall Stage (UICC/AJCC 8), HPV+**					*0.001* ^a^
I	462	60.2	709	63.4	
II	220	28.7	259	23.1	
III	81	10.6	124	11.1	
IV	1	0.1	15	1.3	
Unknown	3	0.4	12	1.1	
**TNM Stage (UICC/AJCC 8), HPV−**					
**T-class**					
T1	105	25.0	30	30.6	*0.74*
T2	138	32.9	32	32.7	
T3	92	21.9	20	20.4	
T4	84	20.0	16	16.3	
Unknown	1	0.2	0		
**N-class**					*0.001* ^a^
N0	162	38.6	21	21.4	
N1	76	18.1	34	34.7	
N2	156	37.1	37	37.8	
N3	24	5.7	6	6.1	
Unknown	2	0.5	0		
**M-class**					*0.001* ^a^
M0	412	98.1	93	94.9	
M1	6	1.4	1	1.0	
Unknown	2	0.5	4	4.1	
**Overall Stage (UICC/AJCC 8), HPV−**					*0.22*
I	74	17.6	18	18.4	
II	71	16.9	9	9.2	
III	72	17.1	18	18.4	
IV	198	47.1	50	51.0	
Unknown	5	1.2	3	3.1	
**Treatment**					*< 0.001* ^a^
RT	368	30.3	188	14.8	
CRT	620	51.0	631	49.8	
Neoadjuvant chemotherapy + RT/CRT	-	-	212	16.7	
Surgery	195	16.0	110	8.7	
Surgery + RT/CRT	33	2.7	124	9.8	
Systemic therapy alone	0	-	2	0.2	
Unspecified curative treatment	0	-	1	0.1	

*Note:* Frequency (%) is provided for categorical variables, median (IQR) are provided for continuous variables. Chi Square test were used for categorical variables, while t-test was used for continuous variables. Fisher’s exact test was used for small sample sizes. *Abbreviations:* UTMDACC, The University of Texas MD Anderson Cancer Center, USA; IQR, interquartile range; HPV, human papillomavirus; BOT, base of tongue; RT, radiotherapy; CRT, chemoradiotherapy.

a Significant value.

**Table 2:** Multi- and univariable analysis for overall survival for OPSCC in Eastern Denmark and UTMDACC from 2015–2020.

	Univariable			Multivariable^c^		
		
Variable	HR	95 % CI	p-value^c^	HR	95 % CI	p-value^b^

**Center (UTMDACC ref)**						
Eastern Denmark	2.20	1.77–2.75	< 0.001^b^	1.21	0.89–1.65	0.23
**Age (<60 years ref)**> 60 years	1.87	1.51–2.32	< 0.001^b^	1.22	0.97–1.55	0.09
**Gender (female ref)**						
Male	1.02	0.79–1.32	0.90	1.30	0.99–1.72	0.06
**HPV status (HPV+ ref)**						
HPV−	4.93	4.00–6.07	< 0.001^b^	2.56	1.92–3.41	< 0.001^b^
**Smoking status (never ref)**						
previous	1.78	1.33–2.39	< 0.001^b^	1.31	0.96–1.79	0.09
current	5.08	3.83–6.73	< 0.001^b^	2.26	1.58–3.22	< 0.001^b^
**Tumor location (tonsil ref)**						
BOT	0.97	0.76–1.22	0.77	0.95	0.74–1.22	0.69
Other	2.62	2.02–3.41	< 0.001^b^	0.86	0.64–1.16	0.33
**T-class (T1 ref)**						
T2	1.42	1.04–1.93	0.03^b^	1.28	0.93–1.77	0.14
T3	3.37	2.46–4.61	< 0.001^b^	2.63	1.86–3.71	< 0.001^b^
T4	3.87	2.84–5.29	< 0.001^b^	2.52	1.78–3.57	< 0.001^b^
**N-class (N0 ref)**						
N1	0.48	0.36–0.64	< 0.001^b^	1.17	0.85–1.65	0.34
N2	1.60	1.21–2.12	0.001^b^	1.88	1.38–2.57	< 0.001^b^
N3	2.92	1.94–4.39	< 0.001^b^	3.79	2.42–5.95	< 0.001^b^
**M-class (M0 ref)**						
M1	3.93	2.26–6.84	< 0.001^b^	4.06	2.26–7.30	< 0.001^b^
**Treatment regimen (CRT ref)**						
RT	2.7	2.15–3.39	< 0.001^b^	2.21	1.71–2.86	< 0.001^b^
Neoajduvant chemotherapy + RT/CRT	1.49	1.04–2.15	0.03^b^	1.31	0.83–2.07	0.24
Surgery	0.70	0.46–1.07	0.10	1.24	0.78–1.98	0.36
Surgery + RT/CRT	0.56	0.31–1.01	0.05	0.89	0.48–1.65	0.71

*Abbreviations:* UTMDACC, The University of Texas MD Anderson Cancer Center, USA; ref, reference; HPV, human papillomavirus, BOT, base of tongue; RT, radiotherapy; CRT, chemoradiotherapy.

b Significant value.

c Adjusted for center, age group, gender, HPV status, smoking status, T-site location, stage UICC8 and treatment regimen.

**Table 3:** Multi- and univariable analysis for recurrence-free interval for OPSCC in Eastern Denmark and UTMDACC from 2015–2020.

	Univariable			Multivariable^e^		
		
Variable	HR	95 % CI	p-value^d^	HR	95 % CI	p-value^d^

**Center (UTMDACC ref)**						
Eastern Denmark	2.11	1.63–2.74	< 0.001^d^	1.74	1.21–2.50	0.003^d^
**Age (<60 years ref)**						
> 60 years	1.53	1.20–1.96	< 0.001^d^	1.08	0.83–1.42	0.56
**Gender (female ref)**						
Male	1.01	0.75–1.38	0.93	1.19	0.86–1.64	0.30
**HPV-status (HPV+ ref)**						
HPV−	3.30	2.58–4.24	< 0.001^d^	2.10	1.50–2.96	< 0.001^d^
**Smoking status (never ref)**						
previous	1.55	1.13–2.12	0.01^d^	1.11	0.80–1.55	0.53
current	3.12	2.27–4.28	< 0.001^d^	1.31	0.88–1.95	0.19
**Tumor location (tonsil ref)**						
BOT	1.04	0.79–1.37	0.76	0.96	0.72–1.28	0.78
Other	2.44	1.77–3.36	< 0.001^d^	1.12	0.78–1.63	0.53
**T-class (T1 ref)**						
T2	1.73	1.20–2.48	0.003^d^	1.71	1.17–2.49	0.005^d^
T3	3.11	2.12–4.58	< 0.001^d^	2.68	1.75–4.09	< 0.001^d^
T4	4.16	2.86–6.04	< 0.001^d^	3.12	2.05–4.75	< 0.001^d^
**N-class (N0 ref)**						
N1	0.73	0.52–1.04	0.08	1.67	1.13–2.47	0.01^d^
N2	2.07	1.45–2.96	< 0.001^d^	2.49	1.66–3.72	< 0.001^d^
N3	3.17	1.85–5.43	< 0.001^d^	4.07	2.27–7.32	< 0.001^d^
**M-class (M0 ref)**				3.22	1.46–7.10	0.004^d^
M1	3.49	1.65–7.41	0.001^d^	3.22	1.46–7.10	0.004^d^
**Treatment regimen (CRT ref)**						
RT	2.19	1.66–2.89	< 0.001^d^	1.98	1.45–2.70	< 0.001^d^
Neoadjuvant chemotherapy + RT/CRT	1.86	1.24–2.77	0.002^d^	2.18	1.32–3.60	0.002^d^
Surgery	0.90	0.58–1.40	0.64	1.64	0.99–2.71	0.05
Surgery + RT/CRT	0.60	0.30–1.18	0.14	0.96	0.46–2.01	0.91

*Abbreviations:* UTMDACC, The University of Texas MD Anderson Cancer Center, USA; ref, reference; HPV, human papillomavirus; BOT, base of tongue; RT, radiotherapy; CRT, chemoradiotherapy.

d Significant value.

e Adjusted for center, age group, gender, HPV status, smoking status, T-site location, stage UICC8 and treatment regimen.
