# Supplementary material for: Clinical and prognostic differences in oropharyngeal squamous cell carcinoma in USA and Denmark, two HPV high-prevalence areas
Source: Eur J Cancer. Author manuscript; Available in PMC 2024 Aug 29. (PMC11357839; doi:10.1016/j.ejca.2024.113983)
Supplement: Appendix A: Supplementary material [file NIHMS2016223-supplement-Appendix_A__Supplementary_material.docx]

**Supplement 1**

1. **Methods and Materials**

*Variables*

The last day of follow-up for the Copenhagen cohort was defined as the last clinical visit, death, or recurrence; and for the UTMDACC cohort as the last imaging, death, or recurrence. Recurrence was defined as local, regional and/or distant. Head and neck cancer within the first 6 months after primary OPSCC was considered progression, i.e., not a recurrence in both cohorts. For the Copenhagen cohort, a new diagnosis of OPSCC identified from six months to five years after primary diagnosis and treatment was considered a recurrence and not a second primary unless specifically stated otherwise in the medical record. For the UTMDACC cohort, the distinction between recurrence or second primary was determined by the treating team based on proximity to the initial cancer and time from treatment (<5 years).

*Measurement data*

Age was grouped as <60 years and >60 years. Patient-reported smoking status at the time of diagnosis was grouped as never smoking/former smoking/current smoking. HPV-positivity was defined as being positive to both p16 immunohistochemistry and HPV DNA in the Copenhagen cohort, while HPV-positivity was defined as being positive to either p16 or HPV DNA/RNA. Clinical stage was defined according to the American Joint Committee on Cancer/Union for International Cancer Control (AJCC/UICC) TNM classification system (8^th^ edition). For p16- tumors, stages IVA-C were merged to IV. Treatment regimen as assigned at a multidisciplinary treatment planning conference and grouped as radiotherapy (RT)/chemoradiotherapy (CRT)/neoadjuvant chemotherapy + RT/CRT/surgery/surgery +RT/CRT. Tumor location was grouped as palatine tonsils/base of tongue/other OPSCC location. Other oropharyngeal sites included in the Copenhagen cohort were pharyngeal wall, soft palate, uvula or pharyngeal arch, while sites included in the UTMDACC cohort were soft palate, pharyngeal wall and glossopharyngeal sulcus.

*HPV detection and p16 immunohistochemistry (IHC)*

For the Copenhagen cohort, HPV DNA was detected with PCR as previously described from 2015-2017^13-15^. From 2018-2020 the presence of HPV DNA and HPV genotypes was evaluated by VisionArray HPV chip 1.0 (ZytoVision). p16 IHC was carried out using Ventana Benchmark Ultra auto-stainer with the UltraView detection kit and the p16 monoclonal antibody E6H4 ready-to-use with CC1 as pretreatment (Roche, Tuscon, USA). The p16-positivity cutoff was set at >70% for both nuclear and cytoplasmatic staining. HPV genotypes was detected with next generation sequencing from 2000-2017 as previously described^13-15^.

For the Stiefel Database, the presence of HPV virus was detected by HPV DNA in situ hybridization, HPV RNA in situ hybridization, cytology genotyping, or HPV DNA PCR. The p16-positivity was assessed by IHC.

*Treatment*

In Denmark, curative radiation-based therapy is the primary treatment and consists of moderately accelerated radiotherapy (RT) given in 33-34 fractions 6 days a week with or without concurrent chemotherapy (CRT). The standard dose levels are 66-68 Gray (Gy) to the clinical target volume (CTV) 1, and 60 Gy to the CTV2. Dose level to elective targets is 50 Gy. According to guidelines, CRT is given based on an evaluation of performance status and comorbidities and consists mainly of weekly cisplatin (40mg/m^2^). Carboplatin is given if cisplatin is not tolerated. Very few patients received cetuximab. Induction therapy is not used in Denmark. Trans-oral robotic surgery (TORS) is considered for patients in clinical trials or if primary RT cannot be performed. In Denmark, all patients were also offered concurrent nimorazole, a radiosensitizer, unless there were contraindications. Unilateral RT could be offered for well-lateralized tonsil carcinomas without involvement of midline structures^16,17^.

At UTMDACC, radiation therapy for OPSCC is generally delivered alone or with concurrent systemic therapy over the course of 6 to 7 weeks (i.e., 33-34 fractions) to a curative total dose of 60-70 Gy. The standard dose levels include 66-70 Gy to CTV1 (i.e., gross tumor with anatomically restricted expansion), 60-63 Gy to adjacent at-risk primary sites, involved neck levels or postoperative volumes, and 54-57 Gy to elective clinical targets (i.e., elective nodal irradiation volumes). Unilateral RT is reserved for well-lateralized clinical T1-T2 tonsil carcinomas with no tongue base involvement, <1cm soft palate involvement, and minimal adenopathy. Concurrent chemotherapy is primarily given as weekly cisplatin (40 mg/m^2^), which can be switched to carboplatin if cisplatin is not tolerated. Currently, few patients who are ineligible to cisplatin receive cetuximab. TORS is considered for all patients with unilateral, low-volume disease (T0-2, N0-1) OPSCC^18^.

*Statistics*

Subgroup analyses were performed for both low-risk patients (T1/T2N0M0) and high-risk patients (stage III-IV). For the T1T2N0M0 analyses the multivariable OS analysis was adjusted for center, HPV-status, smoking status, T-site location, and stage UICC8 based on clinical relevance and due a limited number of events (50). Likewise, the multivariable RFI analysis was adjusted for center, HPV status and stage UICC8 based on clinical relevance and due a limited number of events (26). For the III-IV analyses, the multivariable OS analysis (192 events) was adjusted for center, HPV-status, smoking status, stage UICC8 and treatment regimen while the multivariable RFI (126 events) analysis was adjusted for center, HPV-status, stage UICC8, treatment regimen based on clinical relevance.

1. **Results**

*Subgroup analysis of patients receiving RT as single modality*

The Copenhagen RT single modality cohort comprised significantly more men (27.2% vs 12.2%, p<0.001), more HPV- OPSCC (47.6% vs 9.0%, p<0.001), more other OPSCC location (23.1% vs 5.9%, p<0.001), older age (mean age 68.4 vs 61.3 years, p<0.001), more current smokers (45.1% vs 7.5%, p<0.001) and more stage III-IV (35.9% vs 8.3%, p<0.001). 3-year OS in the Copenhagen RT single modality cohort was 61.6% (95% CI: 56.2-67.5%) and in the UTMDACC RT single modality cohort 88.6% (95% CI: 83.4-94.1%), log-rank p=0.001. Data not shown.

**Table 2. 1- and 3-year overall survival estimates stratified by center and HPV-status from 2015-2020.**

| \| **Overall survival**  **2015-2020** \| **Eastern Denmark** \| **UTMDACC,**  **USA** \| \| --- \| --- \| --- \| \| **OS total** \| % (95% CI) \| % (95% CI) \| \| 1-year \| 92% (91-94%) \| 96% (95-97%) \| \| 3-year \| 83% (80-85%) \| 91% (89-93%) \| \| **OS HPV+** \|  \|  \| \| 1-year \| 96% (94-97%) \| 97% (96-98%) \| \| 3-year \| 88% (86-91%) \| 93% (91-95%) \| \| **OS HPV-** \|  \|  \| \| 1-year \| 85% (81-88%) \| 96% (92-100%) \| \| 3-year \| 56% (51-62%) \| 78% (68-89%) \| |  |  |
| --- | --- | --- | --- | --- | --- | --- | --- | --- | --- | --- | --- | --- | --- | --- | --- | --- | --- | --- | --- | --- | --- | --- | --- | --- | --- | --- | --- | --- | --- | --- | --- | --- |

*Abbreviations:* UTMDACC, The University of Texas MD Anderson Cancer Center, USA; OS, overall survival; HPV, Human Papillomavirus.

**Table 3. Characteristics of 299 patients with T1/T2N0M0 OPSCC in Eastern Denmark and UTMDACC, Texas, USA from 2015-2020.**

|  |  | | | |  |
| --- | --- | --- | --- | --- | --- |
| **Variable** | **Eastern Denmark,**  **n=192** | | **UTMDACC, Texas USA, n=107** | |  |
|  | **no.** | **%** | **no.** | **%** | *p-value** |
| **Gender** |  |  |  |  | *<0.001** |
| Male | 126 | 65.6 | 80 | 74.8 |  |
| Female | 66 | 34.4 | 27 | 25.2 |  |
| **Median age (IQR)** | 65 (59-72) | | 61 (55-67) | | *<0.001** |
| no. | 192 |  | 107 |  |  |
| **Median follow-up. years (IQR)** | 2.2 (1.4-3.2) | | 1.9 (0.8-3.5) | | 0.34 |
| no. | 192 |  | 107 |  |  |
| **Smoking** |  |  |  |  | *<0.001** |
| Current | 108 | 56.3 | 5 | 4.7 |  |
| Former | 61 | 31.8 | 40 | 37.4 |  |
| Never | 22 | 11.5 | 60 | 56.1 |  |
| Unknown | 1 | 0.5 | 2 | 1.9 |  |
| **HPV-status** |  |  |  |  | *<0.001** |
| HPV+ | 69 | 35.9 | 91 | 85.0 |  |
| HPV- | 121 | 63.0 | 14 | 13.1 |  |
| Unknown | 2 | 1.0 | 2 | 1.9 |  |
| **Tumor location** |  |  |  |  | *<0.001** |
| BOT | 24 | 12.5 | 33 | 30.8 |  |
| Tonsils | 84 | 43.8 | 64 | 59.8 |  |
| Other | 84 | 43.8 | 10 | 9.3 |  |
| **TNM Stage (UICC/AJCC 8), HPV+** |  |  |  |  |  |
| **T-class** |  |  |  |  | 1 |
| T1 | 20 | 29.0 | 27 | 29.7 |  |
| T2 | 49 | 71.0 | 64 | 70.3 |  |
| **TNM Stage (UICC/AJCC 8), HPV-** |  |  |  |  |  |
| **T-class** |  |  |  |  | 0.68 |
| I | 55 | 45.5 | 5 | 35.7 |  |
| II | 66 | 54.5 | 9 | 64.3 |  |
| **Overall stage (UICC/AJCC8), HPV-** |  |  |  |  | 0.48 |
| I | 60 | 49.6 | 5 | 35.7 |  |
| II | 61 | 50.4 | 9 | 64.3 |  |
| **Treatment** |  |  |  |  | *<0.001** |
| CRT | 9 | 4.7 | 25 | 23.4 |  |
| RT | 80 | 41.7 | 21 | 19.6 |  |
| Neoadjuvant chemotherapy + RT/CRT | - | - | 1 | 0.9 |  |
| Surgery | 101 | 52.6 | 45 | 42.1 |  |
| Surgery + RT/CRT | 2 | 1 | 15 | 14 |  |
|  |  |  |  |  |  |

*Note:* Frequency (%) is provided for categorical variables, median (IQR) are provided for continuous variables. Chi Square test were used for categorical variables, while t-test was used for continuous variables. Fisher’s exact test was used for small sample sizes.
*Abbreviations:* UTMDACC, The University of Texas MD Anderson Cancer Center, USA; IQR, interquartile range; HPV, Human Papillomavirus; BOT, base of tongue; RT, radiotherapy; CRT, concurrent RT + systemic therapy.

^*^Significant p-value

**Table 4. Multi- and univariable analysis for overall survival for T1/T2N0M0 OPSCC in Eastern Denmark and UTMDACC from 2015-2020.**

|  | **Univariable** | | | **Multivariable**** | | |
| --- | --- | --- | --- | --- | --- | --- |
| **Variable** | **HR** | **95% CI** | **p-value*** | **HR** | **95% CI** | **p-value*** |
| **Center (UTMDACC ref)** |  |  |  |  |  |  |
| Eastern Denmark | 2.63 | 1.23-5.62 | 0.01* | 0.94 | 0.36-2.42 | 0.90 |
| **Age (<60 years ref)** |  |  |  | - |  |  |
| >60 years | 1.83 | 0.55-0.99 | 0.06 |  |  |  |
| **Gender (female ref)** |  |  |  | - |  |  |
| Male | 1.19 | 0.64-2.21 | 0.58 |  |  |  |
| **HPV-status (HPV+ ref)** |  |  |  |  |  |  |
| HPV- | 3.96 | 2.11-7.40 | <0.001* | 1.92 | 0.70-5.21 | 0.20 |
| **Smoking status (never ref)** |  |  |  |  |  |  |
| Previous | 0.96 | 0.36-2.58 | 0.93 | 0.74 | 0.26-2.11 | 0.58 |
| Current | 3.97 | 1.75-9.01 | <0.001* | 2.11 | 0.71-6.27 | 0.18 |
| **Tumor location (tonsils ref)** |  |  |  |  |  |  |
| BOT | 0.81 | 0.30-2.19 | 0.67 | 0.72 | 0.26-2.0 | 0.53 |
| Other | 2.99 | 0.163-5.49 | <0.001* | 1.40 | 0.65-3.0 | 0.39 |
| **UICC stage (I ref)** |  |  |  |  |  |  |
| UICC II | 2.56 | 1.45-4.49 | 0.001* | 0.98 | 0.49-1.94 | 0.94 |
| **Treatment (CRT ref)** |  |  |  | - |  |  |
| RT | 1.73 | 0.66-4.54 | 0.26 |  |  |  |
| Surgery | 0.78 | 0.29-2.1 | 0.62 |  |  |  |
| Surgery + RT/CRT | 0.85 | 0.16-4.36 | 0.84 |  |  |  |

*Abbreviations:* UTMDACC, The University of Texas MD Anderson Cancer Center, USA; ref, reference; HPV, Human Papillomavirus; BOT, base of tongue; RT, radiotherapy; CRT, concurrent RT + systemic therapy. ^*^Significant p-value. **Adjusted for center, HPV-status, smoking status, tumor location and stage UICC8.

**Table 5. Multi- and univariable analysis for recurrence-free interval for T1/T2N0M0 OPSCC in Eastern Denmark and UTMDACC from 2015-2020.**

|  | **Univariable** | | | **Multivariable**** | | |
| --- | --- | --- | --- | --- | --- | --- |
| **Variable** | **HR** | **95% CI** | **p-value*** | **HR** | **95% CI** | **p-value*** |
| **Center (UTMDACC ref)** |  |  |  |  |  |  |
| Eastern Denmark | 5.32 | 1.25-22.6 | 0.02* | 3.14 | 0.67-14.61 | 0.15 |
| **Age (<60 years ref)** |  |  |  | - |  |  |
| >60 years | 2.21 | 0.88-5.53 | 0.09 |  |  |  |
| **Gender (female ref)** |  |  |  | - |  |  |
| Male | 1.30 | 0.54-3.12 | 0.56 |  |  |  |
| **HPV-status (HPV+ ref)** |  |  |  |  |  |  |
| HPV- | 4.10 | 1.63-10.33 | 0.002* | 2.08 | 0.66-6.60 | 0.21 |
| **Smoking status (never ref)** |  |  |  | - |  |  |
| Previous | 1.08 | 0.65-14.51 | 0.15 |  |  |  |
| Current | 5.51 | 1.26-24.17 | 0.02* |  |  |  |
| **Tumor location (tonsils ref)** |  |  |  | - |  |  |
| BOT | 1.50 | 0.44-5.13 | 0.52 |  |  |  |
| Other | 3.27 | 1.31-8.13 | 0.01* |  |  |  |
| **UICC stage (I ref)** |  |  |  | 1.73 | 0.68-4.39 | 0.25 |
| UICC II | 3.19 | 1.45-7.0 | 0.004* |  |  |  |

*Abbreviations:* UTMDACC, The University of Texas MD Anderson Cancer Center, USA; ref, reference; HPV, Human Papillomavirus; BOT, base of tongue. ^*^Significant p-value.** Adjusted for HPV-status, center and stage UICC8.

**Table 6. Characteristics of 560 patients with OPSCC stage III-IV in Eastern Denmark and UTMDACC, Texas, USA from 2015-2020.**

|  |  | | | |  |
| --- | --- | --- | --- | --- | --- |
| **Variable** | **Eastern Denmark,**  **n=353** | | **UTMDACC, Texas USA, n=207** | |  |
|  | **no.** | **%** | **no.** | **%** | *p-value** |
| **Gender** |  |  |  |  | *<0.001** |
| Male | 260 | 73.7 | 190 | 91.8 |  |
| Female | 93 | 26.3 | 17 | 8.2 |  |
| **Median age (IQR)** | 64 (58-70) | | 63 (57-68) | | *0.02** |
| no. | 353 |  | 207 |  |  |
| **Median follow-up. years (IQR)** | 1.9 (1.2-2.8) | | 2.1 (1.1-3.6) | | *0.02** |
| no. | 192 |  | 207 |  |  |
| **Smoking** |  |  |  |  | *<0.001** |
| Current | 221 | 62.6 | 19 | 9.2 |  |
| Former | 104 | 29.5 | 106 | 51.2 |  |
| Never | 28 | 7.9 | 81 | 39.1 |  |
| Unkown | 0 |  | 1 | 0.5 |  |
| **HPV-status** |  |  |  |  | *<0.001** |
| HPV+ | 82 | 23.2 | 139 | 67.1 |  |
| HPV- | 270 | 76.5 | 68 | 32.9 |  |
| Unknown | 1 | 0.3 | 0 | - |  |
| **Tumor location** |  |  |  |  | *<0.001** |
| BOT | 137 | 38.8 | 127 | 61.4 |  |
| Tonsils | 114 | 32.3 | 66 | 31.9 |  |
| Other | 102 | 28.9 | 14 | 6.8 |  |
| **TNM Stage (UICC/AJCC 8), HPV+** |  |  |  |  |  |
| **T-class** |  |  |  |  | 0.86 |
| T1 | 8 | 9.8 | 10 | 7.2 |  |
| T2 | 9 | 11.0 | 13 | 9.4 |  |
| T3 | 30 | 36.6 | 56 | 40.3 |  |
| T4 | 35 | 42.7 | 60 | 43.2 |  |
| **N-class** |  |  |  |  | *0.002** |
| N0 | 0 | - | 1 | 0.7 |  |
| N1 | 0 | - | 4 | 2.9 |  |
| N2 | 53 | 64.6 | 112 | 80.6 |  |
| N3 | 29 | 35.4 | 22 | 15.8 |  |
| **M-class** |  |  |  |  | *0.003** |
| M0 | 79 | 96.3 | 125 | 89.9 |  |
| M1 | 1 | 1.2 | 14 | 10.1 |  |
| Unkown | 2 | 2.4 | 0 | - |  |
| **Overall stage (UICC/AJCC8), HPV+** |  |  |  |  | *0.007** |
| III | 81 | 98.8 | 124 | 89.2 |  |
| IV | 1 | 1.2 | 15 | 10.8 |  |
| **TNM Stage (UICC/AJCC 8), HPV-** |  |  |  |  |  |
| **T-class** |  |  |  |  | 0.57 |
| T1 | 45 | 16.7 | 11 | 16.2 |  |
| T2 | 63 | 23.3 | 22 | 32.4 |  |
| T3 | 83 | 30.7 | 19 | 27.9 |  |
| T4 | 79 | 29.3 | 16 | 5.9 |  |
| **N-class** |  |  |  |  | 0*.*44 |
| N0 | 37 | 13.7 | 6 | 8.8 |  |
| N1 | 54 | 20 | 19 | 27.9 |  |
| N2 | 155 | 57.4 | 37 | 54.4 |  |
| N3 | 24 | 8.9 | 6 | 8.8 |  |
| **M-class** |  |  |  |  | 0.23 |
| M0 | 264 | 97.8 | 66 | 97.1 |  |
| M1 | 6 | 2.2 | 1 | 1.5 |  |
| Unknown | 0 | - | 1 | 1.5 |  |
| **Overall stage (UICC/AJCC8), HPV-** |  |  |  |  | 1 |
| III | 72 | 26.7 | 18 | 26.5 |  |
| IV | 198 | 73.3 | 50 | 73.5 |  |
| **Treatment** |  |  |  |  | *<0.001** |
| CRT | 201 | 56.9 | 103 | 49.8 |  |
| RT | 132 | 37.4 | 15 | 7.2 |  |
| Neoadjuvant chemotherapy + RT/CRT |  |  | 81 | 39.1 |  |
| Surgery | 12 | 3.4 | 1 | 0.5 |  |
| Surgery RT/CRT | 8 | 2.3 | 7 | 3.4 |  |

*Note:* Frequency (%) is provided for categorical variables, median (IQR) are provided for continuous variables. Chi Square test were used for categorical variables, while t-test was used for continuous variables. Fisher’s exact test was used for small sample sizes.  *Abbreviations:* UTMDACC, The University of Texas MD Anderson Cancer Center, USA; IQR, interquartile range; HPV, Human Papillomavirus; BOT, base of tongue; RT, radiotherapy; CRT, concurrent RT + systemic therapy.

^*^Significant p value

**Table 7. Multi- and univariable analysis for overall survival for OPSCC UICC8 Stage III/IV in Eastern Denmark and UTMDACC from 2015-2020.**

|  | **Univariable** | | | **Multivariable**** | | |
| --- | --- | --- | --- | --- | --- | --- |
| **Variable** | **HR** | **95% CI** | **p-value*** | **HR** | **95% CI** | **p-value*** |
| **Center (UTMDACC ref)** |  |  |  |  |  |  |
| Eastern Denmark | 2.51 | 1.76-3.57 | <0.001* | 2.20 | 1.29-3.75 | 0.004* |
| **Age (<60 years ref)** |  |  |  | - |  |  |
| <60 years | 1.07 | 0.79-1.43 | 0.68 |  |  |  |
| **Gender (female ref)** |  |  |  | - |  |  |
| Male | 0.93 | 0.66-1.31 | 0.67 |  |  |  |
| **HPV-status (HPV+ ref)** |  |  |  |  |  |  |
| HPV- | 1.91 | 1.39-2.63 | <0.001* | 0.76 | 0.46-1.26 | 0.29 |
| **Smoking status (never ref)** |  |  |  |  |  |  |
| Previous | 1.88 | 1.14-3.12 | 0.01* | 1.52 | 0.89-2.58 | 0.23 |
| Current | 3.00 | 1.85-4.85 | <0.001* | 1.85 | 1.03-3.30 | 0.04* |
| **Tumor location (tonsils ref)** |  |  |  | - |  |  |
| BOT | 0.74 | 0.53-1.03 | 0.07 |  |  |  |
| Other | 1.06 | 0.74-1.52 | 0.74 |  |  |  |
| **UICC stage (III ref)** |  |  |  |  |  |  |
| UICC IV | 1.85 | 1.39-2.48 | <0.001* | 1.80 | 1.24-2.62 | 0.002* |
| **Treatment (CRT ref)** |  |  |  |  |  |  |
| RT | 2.34 | 1.72-3.19 | <0.001* | 2.20 | 1.59-3.04 | <0.001* |
| Neoadjuvant chemotherapy + RT/CRT | 0.84 | 0.52-1.35 | 0.47 | 1.84 | 0.96-3.51 | 0.07 |
| Surgery | 0.83 | 0.30-2.32 | 0.72 | 0.75 | 0.26-2.14 | 0.59 |
| Surgery + RT/CRT | 0.86 | 0.35-2.11 | 0.74 | 1.07 | 0.42-2.72 | 0.88 |

*Abbreviations:* UTMDACC, The University of Texas MD Anderson Cancer Center, USA; ref, reference; HPV, Human Papillomavirus; BOT, base of tongue; RT, radiotherapy; CRT, concurrent RT + systemic therapy. ^*^significant p-value. **Adjusted for center, HPV-status, smoking status and treatment regimen.

**Table 8. Multi- and univariable analysis for recurrence-free interval for OPSCC UICC8 Stage III/IV in Eastern Denmark and UTMDACC from 2015-2020.**

|  | **Univariable** | | | **Multivariable**** | | |
| --- | --- | --- | --- | --- | --- | --- |
| **Variable** | **HR** | **95% CI** | **p-value*** | **HR** | **95% CI** | **p-value*** |
| **Center (UTMDACC ref)** |  |  |  |  |  |  |
| Eastern Denmark | 1.95 | 1.30-2.93 | 0.001* | 2.80 | 1.46-5.38 | 0.002* |
| **Age (<60 years ref)** |  |  |  | - |  |  |
| >60 years | 0.97 | 0.67-1.38 | 0.85 |  |  |  |
| **Gender (female ref)** |  |  |  | - |  |  |
| Male | 0.95 | 0.62-1.46 | 0.81 |  |  |  |
| **HPV-status (HPV+ ref)** |  |  |  |  |  |  |
| HPV- | 1.52 | 1.04-2.22 | 0.03* | 0.74 | 0.41-1.33 | 0.32 |
| **Smoking status (never ref)** |  |  |  | - |  |  |
| Previous | 1.11 | 0.67-1.88 | 0.67 |  |  |  |
| Current | 1.48 | 0.90-2.44 | 0.12 |  |  |  |
| **Tumor location (tonsils ref)** |  |  |  | - |  |  |
| BOT | 0.92 | 0.61-1.40 | 0.70 |  |  |  |
| Other | 1.46 | 0.94-2.270 | 0.09 |  |  |  |
| **UICC stage (III ref)** |  |  |  |  |  |  |
| UICC IV | 1.92 | 1.34-2.74 | <0.001* | 2.20 | 1.35-3.57 | 0.001* |
| **Treatment (CRT ref)** |  |  |  |  |  |  |
| RT | 1.90 | 1.28-2.81 | 0.001* | 1.84 | 1.22-2.76 | 0.004* |
| Neoadjuvant chemotherapy + RT/CRT | 1.29 | 0.77-2.15 | 0.33 | 3.10 | 1.45-6.65 | 0.004* |
| Surgery | 0.66 | 0.16-2.71 | 0.57 | 0.72 | 0.17-2.98 | 0.65 |
| Surgery + RT/CRT | 0.81 | 0.25-2.60 | 0.73 | 1.13 | 0.34-3.70 | 0.85 |
|  |  |  |  |  |  |  |

*Abbreviations:* UTMDACC, The University of Texas MD Anderson Cancer Center, USA; ref, reference; HPV, Human Papillomavirus; BOT, base of tongue; RT, radiotherapy; CRT, concurrent RT + systemic therapy. ^*^Significant p-value. ** Adjusted for center, HPV-status, stage UICC8, treatment regimen.

**Figure 1. Kaplan-Meier curves depicting the overall survival and recurrence-free interval stratified by center for T1/T2N0M0 OPSCC from 2015-2020.** A. Overall survival. B. Recurrence-free interval.

**A.**


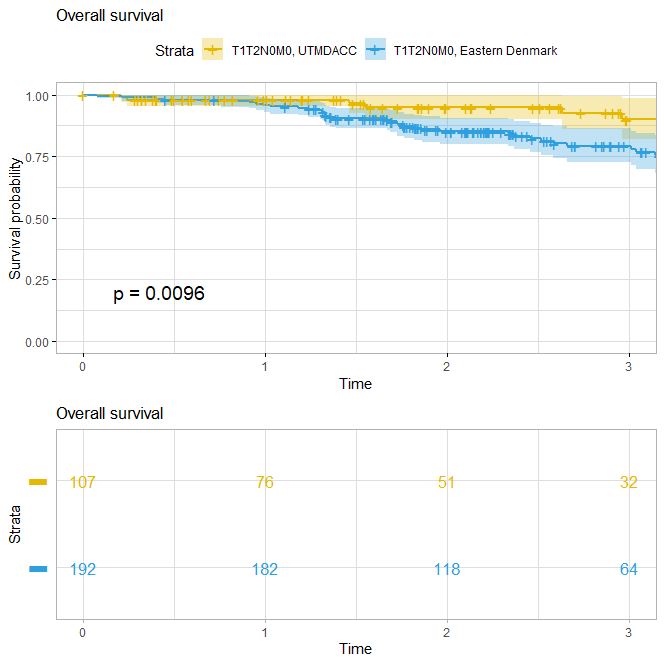


**B.**


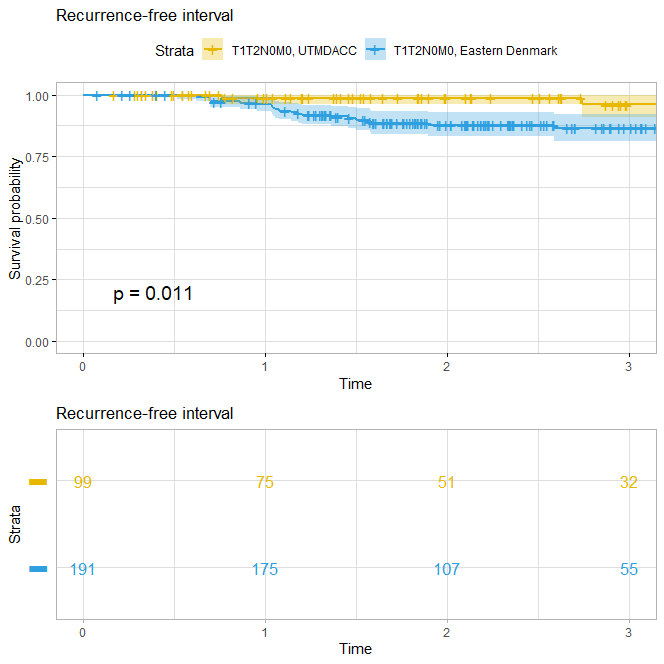


*Abbreviations:* UTMDACC, The University of Texas MD Anderson Cancer Center, USA, HPV, Human Papillomavirus; OPSCC, oropharyngeal squamous cell carcinoma.

**Figure 2. Kaplan-Meier curves depicting the overall and recurrence-free interval stratified by center for OPSCC UICC8 stage III-IV from 2015-2020.** A. Overall survival. B. Recurrence-free interval.

**A.**


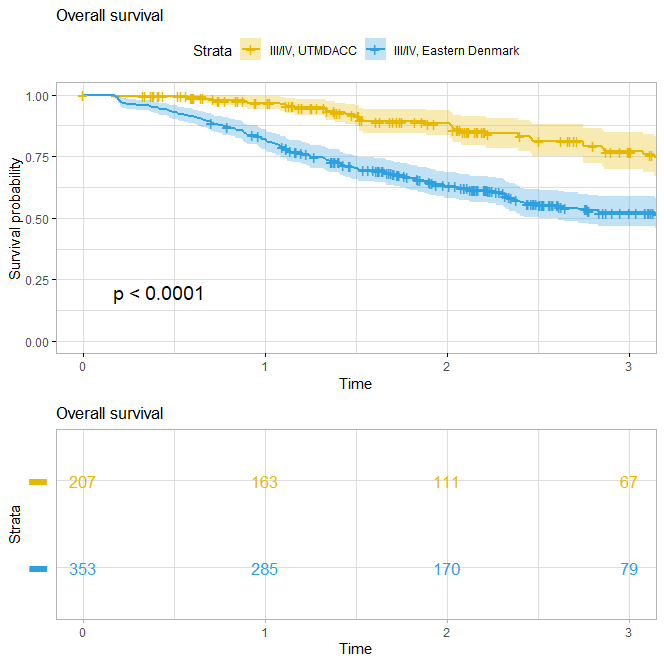


**B.**


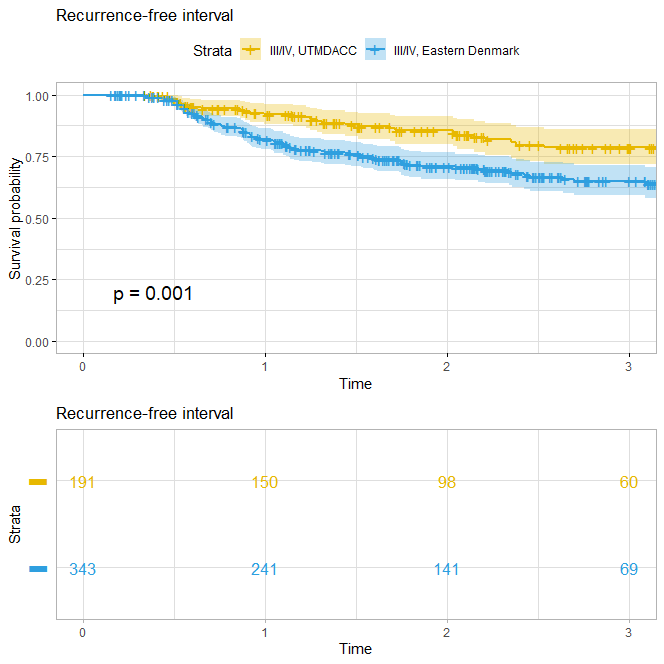


*Abbreviations:* UTMDACC, The University of Texas MD Anderson Cancer Center, USA, HPV, Human Papillomavirus; OPSCC, oropharyngeal squamous cell carcinoma.
